# Adoption of Individual Flood Damage Mitigation Measures in New York City: An Extension of Protection Motivation Theory

**DOI:** 10.1111/risa.13318

**Published:** 2019-04-25

**Authors:** W. J. Wouter Botzen, Howard Kunreuther, Jeffrey Czajkowski, Hans de Moel

**Affiliations:** ^1^ Institute for Environmental Studies VU University Amsterdam The Netherlands; ^2^ Utrecht University School of Economics Utrecht University Utrecht The Netherlands; ^3^ Risk Management and Decision Processes Center, The Wharton School University of Pennsylvania Philadelphia USA

**Keywords:** Charity hazard, flood risk mitigation, protection motivation theory, risk aversion, time preferences

## Abstract

This study offers insights into factors of influence on the implementation of flood damage mitigation measures by more than 1,000 homeowners who live in flood‐prone areas in New York City. Our theoretical basis for explaining flood preparedness decisions is protection motivation theory, which we extend using a variety of other variables that can have an important influence on individual decision making under risk, such as risk attitudes, time preferences, social norms, trust, and local flood risk management policies. Our results in relation to our main hypothesis are as follows. Individuals who live in high flood risk zones take more flood‐proofing measures in their home than individuals in low‐risk zones, which suggests the former group has a high threat appraisal. With regard to coping appraisal variables, we find that a high response efficacy and a high self‐efficacy play an important role in taking flood damage mitigation measures, while perceived response cost does not. In addition, a variety of behavioral characteristics influence individual decisions to flood‐proof homes, such as risk attitudes, time preferences, and private values of being well prepared for flooding. Investments in elevating one's home are mainly influenced by building code regulations and are negatively related with expectations of receiving federal disaster relief. We discuss a variety of policy recommendations to improve individual flood preparedness decisions, including incentives for risk reduction through flood insurance, and communication campaigns focused on coping appraisals and informing people about flood risk they face over long time horizons.

## 1 INTRODUCTION

Costly flood events around the world and expectations that flood risk can rise in many regions as a result of climate change have increased the importance of effective flood risk management policies (IPCC, 2013, 2014). For example, the 2017 hurricane season was a record in terms of economic costs, in which Hurricane Harvey resulted in flood damage in the United States alone of $85 billion.^1^

A traditional method for preventing flood damage is to install public flood protection measures, like dike infrastructure or stormwater detention and retention basins. In addition, flood damage mitigation measures for individual properties can minimize the impacts of flood events, such as elevating homes or flood‐proofing buildings. Several studies provide evidence that such measures can significantly limit damage during flood events, and are also cost effective in areas with a high flood risk (Aerts et al., 2014; Kreibich, Thieken, Petrow, Müller, & Merz, 2005, 2011; Poussin, Botzen, & Aerts, 2015).

Despite these advantages, many people in flood‐prone areas are not well prepared for flood events because they do not implement cost‐effective flood damage mitigation measures (Bubeck, Botzen, Kreibich, & Aerts, 2013; Kunreuther, 1996; Meyer, Baker, Broad, Czajkowski, & Orlove, 2014). As an illustration, a survey of coastal residents in New Jersey, Delaware, Maryland, and Virginia, ahead of the landfall of Hurricane Sandy, revealed that only about 25% of 593 respondents had modified their home to reduce the amount of damage from a hurricane (Meyer et al., 2014). Hence, it is important to understand the determinants of flood preparedness decisions because such insights can guide the design of policies that help people make better decisions in preparing for flood events.

This study examines the implementation of flood damage mitigation measures by homeowners who live in flood‐prone areas in New York City (NYC) in order to identify factors that influence individual flood preparedness decisions. For this purpose, we collected data by conducting a survey, which has been completed by more than 1,000 homeowners. This survey included questions about whether or not individuals had elevated their home or implemented a range of dry flood‐proofing measures to prevent floodwaters from entering a building, and utilized wet flood‐proofing measures to reduce damage once floodwater has entered a building. The survey was conducted shortly after NYC was flooded by Hurricane Sandy in 2012, which caused about $19 billion of damage to the city alone (NYC, 2013). These high damages caused by Hurricane Sandy highlight the importance of improving individual flood preparedness in NYC in order to minimize damages of future flood events. It is thus important to understand why some homeowners in NYC decided to implement flood damage mitigation measures, while others had not done so.

Our theoretical basis for explaining flood mitigation decisions is protection motivation theory that was developed in the 1970s to explain when individuals would undertake preventive measures to reduce their health‐related risk (Floyd, Prentice‐Dunn, & Rogers, 2000; Milne et al., 2000; Rogers, 1975). More recently it has been applied in several studies to explain flood preparedness decisions in various countries, including Germany (Bubeck et al., 2013; Grothmann & Reusswig, 2006), Scotland (Glenk & Fischer, 2010), Vietnam (Reynaud, Aubert, & Nguyen, 2013), France (Poussin, Botzen, & Aerts, 2014), and Australia (Franklin, King, Aitken, & Leggat, 2014). Protection motivation theory explains individual decisions about preparing for risk on the basis of threat appraisal and coping appraisal, which include the perceived effectiveness, ability to implement, and costs of protective measures. For instance, in the context of flood risk, threat appraisal captures individual flood risk perceptions, and coping appraisal captures the degree to which an individual finds a flood‐proofing measure effective, easy to implement, and not too costly. Even though these variables have been shown to explain protective behavior in a variety of contexts (Bubeck, Botzen, & Aerts, 2012a), several other factors such as risk attitudes, time preferences, social norms, and public‐sector flood risk management policies may also influence flood preparedness decisions.

Theories of individual decision making under risk have pointed toward the importance of risk attitudes and time preferences in determining individual demand for protective investments (e.g., Loewenstein & Prelec, 1992). In addition to these variables, social norms may also influence decision making under risk (Elster, 1989). For instance, it has been shown that people are more likely to undertake measures to prepare for flooding if they know others, like neighbors and friends, have also implemented flood preparedness measures or if they discussed such measures with them (Bubeck et al., 2013; Kunreuther, 1978).

The public sector can play a role in determining whether individuals will invest in loss reduction measures. Well‐enforced standards can also influence individual flood mitigation and preparedness decisions, such as building codes that require the elevation of newly constructed buildings in high‐risk flood zones as is the case in NYC (Aerts & Botzen, 2011). After a severe natural disaster, the U.S. federal government may provide partial compensation to households to aid their recovery process, which may lower their economic incentives to undertake measures to limit flood risk (Kousky, Michel‐Kerjan, & Raschky, 2018). In our analyses, we extend the basic protection motivation theory framework to include these additional variables to arrive at a more complete picture of factors that influence the implementation of flood damage mitigation measures.

The remainder of this article is structured as follows. Section 2 discusses the survey and statistical methods, data, and key hypotheses of interest. Section 3 presents the results in terms of descriptive statistics of implemented flood damage mitigation measures and statistical models of factors of influence on the implementation of these measures. Section 4 discusses the main results in relation to the hypotheses and findings from other studies, and offers policy recommendations. Section 5 concludes.

## 2 METHODS, DATA, AND HYPOTHESES

### 2.1 Survey

A telephone survey was conducted by randomly calling homeowners who reside in a house with a ground floor in flood‐prone areas of NYC. Two focus group were organized in 2012 with NYC experts involved in flood risk management to give feedback on the sample selection and design of the survey. The survey questions were pretested in another focus group with NYC residents, and the final phone survey was pretested with 73 people (including 35 NYC residents).^2^ The final survey was implemented between March and April 2013 by 35 professional and trained interviewers of the company Kerr and Downs Research, using computer‐assisted telephone interviewing. Of the qualified respondents, 73% completed the survey, which resulted in 1,035 observations. The high response rate indicates that many floodplain inhabitants were willing to participate in the survey on this theme after NYC experienced flooding from Hurricane Sandy.

### 2.2 Dependent Variables of Flood Damage Mitigation Measures

Several survey questions asked respondents whether they implemented specific flood risk mitigation measures and, if so, whether this measure was in place before or after the most recent flood they experienced. Three dependent variables were created on the basis of these answers: a binary variable indicating whether people elevated their home above the expected flood water level or not, and variables on the number of dry and wet flood‐proofing measures people undertook. A distinction between these measures is made because elevation is required for new construction in the 1/100‐year flood zone according to the NYC building code regulations, while dry and wet flood‐proofing measures are not required for residential buildings (Aerts & Botzen, 2011). Dry and wet flood‐proofing measures each have a different purpose. In particular, dry flood‐proofing measures aim to keep water out of a building during a flood. They include water‐proofing walls, installing flood shields or sand bags, and having a pump or drainage system. Wet flood‐proofing measures aim to limit damage once water has entered the building. They include building with flood‐resistant building materials, having a water‐resistant floor, placing utility and electricity installations above potential flood levels, and keeping furniture or contents out of flood‐prone parts of the house.

Aerts, Botzen, de Moel, and Bowman (2013a) have estimated the costs of taking these flood damage mitigation measures for residential homes in NYC, which are reported in Table I as unit cost and for a single residential home. The latter are given as a range since the costs depend on the type of residential structure. Costs are shown for implementing the measures +2 feet above the baseline flood elevation (BFE), the expected water level of the 1/100‐year flood event. The following main pattern appears from the cost figures described in more detail in Aerts et al. (2013a). Elevation is relatively cheap when it is conducted during construction (up to $7,404), but very expensive when an existing building is elevated (up to $91,732). Moreover, elevation costs vary substantially depending on the existing grade elevation compared with the BFE as well as on the nature of the foundation and structural type (for more detail, see Aerts et al., 2013a). Dry and wet flood‐proofing existing homes are considerably cheaper than elevation, and of the flood‐proofing measures, wet flood‐proofing is slightly cheaper than dry flood‐proofing. Only aggregate cost figures are provided for wet flood‐proofing measures because a detailed breakdown of cost per individual measure is lacking. These wet flood‐proofing costs include adding wall openings for the entry and exit of floodwaters, installing pumps, rearranging or relocating utility systems, moving large appliances, and facilitating clean up after floodwaters recede (Aerts et al., 2013a).

**Table I:** Average Costs of Elevating and Implementing Dry and Wet Flood‐Proofing Measures for a Residential Home in New York City

	Unit Cost	Cost for a Single Home
Elevating		
Elevating existing home +2 feet above BFE	$85 per sq. ft. of building footprint	$44,208 up to $91,732
Elevating newly built home +2 feet above BFE	$5 per sq. ft. of building footprint	$1,450 up to $7,404
Dry flood‐proofing measures		
Water‐resistant wall	$12 per linear ft. of wall	
Drainage	$41 per linear ft.	
Pump	$2,274	
Flood shields	$499 per linear ft.	
Total dry proofing +2 feet		$11,026 up to $21,126
Wet flood‐proofing	$2.20 up to $2.90 per sq. ft. of building footprint	$2,861 up to $19, 307

*Source*: Aerts et al. (2013a).

Aerts et al. (2014) present cost–benefit analysis results of elevating and dry and wet flood‐proofing residential homes in the 1/100‐year flood zones in NYC.^3^ These estimates reflect the higher benefits of flood risk mitigation from climate change scenarios of sea‐level rise and hence the potential increases in hurricane probabilities and the resulting flood risk. Here, we summarize the main results of the most likely risk estimates in Aerts et al. (2014). A range is provided, which reflects uncertainty about the climate change scenario, discount rate, and effectiveness of flood proofing. Elevation of new structures +2 feet above the BFE is cost effective with benefit–cost ratios ranging between 1.80 and 37.48, while elevation of existing buildings is not economically desirable since benefit–cost ratios range between 0.1 and 0.59. On the other hand, wet flood‐proofing existing buildings is more cost effective than elevating the structures, with benefit–cost ratios ranging between 0.45 and 4.78, while those of dry flood‐proofing range between 0.37 and 2.65. In summary, for new buildings, elevation is economically the most attractive method, which is consistent with NYC building code regulations, which require elevation of new structures in the 1/100‐year flood zone. For existing buildings flood‐proofing measures, notably wet flood‐proofing, are more cost effective than elevating the structures.

### 2.3 Explanatory Variables and Hypotheses

We take protection motivation theory, which was introduced and revised by Rogers (1975, 1983), as a starting point for our model. This theory explains protective behavior of individuals according to two cognitive processes: namely, “threat appraisal” and “coping appraisal.” Threat appraisal describes how an individual evaluates how threatened he or she feels by a certain risk, which has also been referred to as “risk perception” (Grothmann & Reusswig, 2006) in the spirit of the research undertaken by Slovic and his colleagues (Slovic, 2000). Only an objective flood risk indicator *R_i_* is included in our models as proxy for threat appraisal, and not indicators of flood risk perceptions. The reason is that cross‐sectional survey data cannot unambiguously identify the relationship between flood risk perceptions and the decision on whether to invest in flood‐proofing measures, as pointed out by other studies (Bubeck et al., 2012a; Siegrist, 2013).^4^

Objective flood risk is approximated by the FEMA flood zones.^5^ In particular, we include a dummy variable of respondents living in the 1/100‐year flood zone to examine whether these people adopt more flood risk mitigation measures compared to those residing in the lower‐risk flood zone (the X zone). We recognize that this variable is only indirectly related to threat appraisal,^6^ but note that 86% of our respondents responded that they are aware that they are living in a flood‐prone area, implying that they are knowledgeable about their flood risk.

**H1**: We hypothesize that individuals who live in high FEMA flood risk zones have undertaken more flood damage mitigation measures than individuals in the low FEMA flood risk zone.

Flood risk may also be more salient for those who personally experienced flooding (variable *E_i_*) and this experience is likely to increase their threat appraisal and uptake of flood damage mitigation measures.^7^ In this regard, Osberghaus (2017) shows, using survey data of a representative sample of German households, that flood experience is positively related to the implementation of flood risk mitigation measures. We thus hypothesize that a similar relationship exists in our data, although we expect that flood experience will be less important in explaining adoption of mitigation measures in our survey since all the respondents resided in an area affected by an extreme flood event, while the sample studied by Osberghaus (2017) had substantial heterogeneity in their flood experiences. We defined households with flood experience as respondents who answered that their current household had been previously affected by floods caused by natural disasters at least once.^8^

**H2**: We hypothesize that individuals who experienced a flood in their home have taken more flood damage mitigation measures than individuals without flood experience.

Once a certain level of threat appraisal is reached, people start to think about the benefits of possible actions and to evaluate their own competence to carry them out. This process is referred to as “coping appraisal,” *Ci*, and is included as three separate explanatory variables “response efficacy,” “self‐efficacy,” and “response cost.” Our survey measured these three variables separately for each of our three categories of flood risk mitigation measures. *Response efficacy* addresses to what extent an individual believes that a protective measure effectively reduces a risk. *Self‐efficacy* reflects the belief of a person as to whether he or she is personally able to actually carry out the specific measure. *Response costs* are the person's estimate of how costly it would be for him or her to actually implement the particular risk‐reduction measure, including perceived time, effort, and the costs of implementing the measure.^9^

**H3**: We hypothesize that perceptions of a high response efficacy, a high self‐efficacy, and low response cost are positively related with the implementation of flood damage mitigation measures.

The standard protection motivation theory framework does not capture several other potentially important behavioral variables (*B_i_*) that are likely to influence flood preparedness decisions, such as discounting, risk aversion, norms of preparing for flooding, expectations of federal disaster relief, and trust in NYC flood risk management. We add these variables separately to the model.

**H4**: With respect to *discounting*, we hypothesize that individuals with a high discount rate take less flood damage mitigation measures.

The reason is that individuals may not invest in flood risk mitigation measures that have high upfront investment cost if they heavily discount the future benefits of reduced risk in their investment decision (Kunreuther, Pauly, & McMorrow, 2012). To determine discounting by individuals, we elicit individual time preferences (related to discounting) using a question format proposed by Falk, Becker, Dohmen, Huffman, and Sunde (2012) “When it comes to financial decisions, how would you assess your willingness to give up something today in order to benefit from that in the future?” with answer categories 1 = *completely unwilling to give up something today* up to 10 = *very willing to give up something today*. Falk et al. (2012) test the validity of four different survey questions to elicit individual time preferences by comparing how answers predict an incentivized experiment for eliciting individual discount rates, and concluded that the format we use has the best explanatory power.

A similar format was used to elicit individual *risk aversion* using the following survey question: “Using a 10‐point scale where 1 means you are not willing to take any risks and 10 means you are very willing to take risks, what number reflects how much risk you are willing to take?” This question is based on Dohmen et al. (2011), who show that this question has behavioral validity in predicting risk attitudes in an experiment using paid lotteries. Moreover, the question appears to be a good predictor of a wide range of risky behavior, such as willingness to take risks in car driving, financial matters, sports/leisure, career, and health (Dohmen et al., 2011).

**H5**: We hypothesize that individuals with a low level of risk aversion are less likely to take flood damage mitigation measures.

*Norms and values* may be a motivation for people to prepare for disasters and undertake flood risk mitigation measures. Being adequately prepared for a specific risky situation may be regarded as a social norm, so that households do not need to rely on others for assistance during and after a disaster. The importance of distinguishing between different types of social norms has been found in a variety of contexts such as littering, recycling, and energy savings (see the review in Huber, Viscusi, & Bell, 2017). For instance, in the context of individual recycling decisions, Viscusi, Huber, and Bell (2011) show that it is important to distinguish between a social norm that affects a person's behavior due to the actions of others and private values because they find the former did not influence recycling while private values did. In our study, a social norm refers to approval of others of being well prepared for flooding, while a private value refers to behavior that the respondent finds to be personally important. The private value was measured using the question: “Please tell me if you strongly agree, agree, neither agree nor disagree, disagree, or strongly disagree with the following statement: I would be upset if I noticed that someone who got flooded was insufficiently prepared for flooding and needed to request federal compensation for flood damage he suffered.” For eliciting the social norm, the text was: “Other people would be upset if they noticed that someone who got flooded was insufficiently prepared for flooding and needed to request federal compensation for flood damage he suffered.” The private value and social norm variables take on the value 1 if the respondent agreed or strongly agreed with the relevant statements and 0 otherwise. In line with the results about social norms and private values found by Viscusi et al. (2011), we draw the following hypotheses:

**H6**: We hypothesize that a strong private value of being well prepared for flooding is positively related to implemented flood damage mitigation measures.
**H7**: We hypothesize that a strong social norm of being well prepared for flooding is not significantly related with implemented flood damage mitigation measures.

Individuals who expect to receive compensation for flood damage from the federal government may be less likely to take measures themselves to limit flood risk, which has been called the charity hazard effect (Raschky & Weck‐Hanneman, 2007). Charity hazard may be an issue in the United States because often federal relief is provided to victims of flood disasters. There is an element of uncertainty involved *ex ante* about whether or not and how much relief will be obtained and many individuals perceive that they will obtain much more federal assistance than they actually will obtain if they experience flood damage (Kousky et al., 2018). We measure expectations of receiving federal disaster relief in the survey by asking for the percentage of damage a respondent expects to be compensated by the federal government in case a flood occurs (which is 0% for respondents who do not expect any compensation).

**H8**: We hypothesize that high expectations of receiving federal disaster relief are negatively related to the implementation of flood damage mitigation measures.

Elevation of newly constructed residential buildings is the main flood risk mitigation measure for households that is advocated by NYC flood risk management policies. The NYC building code stipulates that flood‐proofing measures can be used for commercial buildings to meet flood‐resistant building regulations, but not for residential buildings for which elevation is the only means to comply with the building code (Aerts & Botzen, 2011).

**H9**: Hence, it can be expected that individuals with a high trust in NYC flood risk management policies, which advocate elevation, are more likely to have elevated their home above potential flood water levels.

In addition to our main interests of testing H1–H9, we include a variety of control variables of home characteristics *Hi* and sociodemographic characteristics *Xi*. The former include whether or not the home was built after 1986, which is when new constructions in NYC became subject to requirements to elevate the lowest floor of buildings above the potential water level of the 1 in 100‐year flood,^10^ and whether or not the home has a basement. Homes with a basement are considered more vulnerable to suffering flood damage, which makes it more attractive to install wet or dry flood‐proofing measures. Sociodemographic variables include gender, education level, and age.

### 2.4 Statistical Methods

We estimate separate statistical models of factors influencing the decision to invest in each of our three categories of flood risk mitigation measure, since different behavioral motivations may underlie decisions to undertake each of these measures. For instance, a main reason for elevating new homes may be to comply with NYC building codes, while property owners who aim to keep floodwaters out of their buildings undertake dry flood‐proofing measures. Those who want to limit damage once water has entered a building invest in wet flood‐proofing measures. Preferences for, and perceived effectiveness of, dry and wet flood‐proofing measures can differ between residents, which is why they are elicited separately and included in separate statistical models.

The general specification for each of these three models takes the following form:

(1) $$\begin{array}{ccc} Y{\left(\mathrm{risk}\;\mathrm{mitigation}\right)}_{i} & = & \beta_{1}+\alpha_{1}R_{i}+\lambda_{1}E_{i}+\gamma_{1}C_{i}\\ & & +\;\phi_{1}B_{i}+\delta_{1}H_{i}+\mu_{1}X_{i}+\varepsilon_{i}, \end{array}$$

where the dependent variable *Y_i_* is either the binary variable of home elevation or count variable of the number of dry or wet flood‐proofing measures implemented by individual *i*. *R_i_* is a flood risk indicator, which is a proxy of threat appraisal; *E_i_* is flood experience, which indicates the saliency of flood risk that can influence threat appraisal; and *C_i_* is a vector of coping appraisal variables. The extensions of the protection motivation theory framework are captured by *B_i_*, which are behavioral characteristics of the respondents that can influence the uptake of flood risk mitigation measures, *H_i_*, which are home characteristics, and *Xi*, which are sociodemographic characteristics of the respondent. $\varepsilon_{i}$ is the error term. Our main hypotheses are tested by estimating the full regression models indicated by Equation (1). In addition, we examine whether the results are robust by estimating models using a step‐wise regression method that excludes insignificant variables (*p*s > 0.1) one by one (Wooldridge, 2002).^11^ We show results for full models that either include the private value or social norm variable because these variables are strongly correlated and including them together in a single model results in problems with multicollinearity. Table AI in the Supporting Information gives the description and coding of each of the variables.^12^ The dependent variable of home elevation is binary since the home is either elevated or not, which is why a probit model is employed as an estimation method for that measure. The dependent variables of the number of implemented dry and wet flood‐proofing measures are count variables of the number of implemented measures of that category, for which Poisson regression models are most appropriate to employ as estimation method.

## 3 RESULTS

### 3.1 Descriptive Statistics of Implemented Flood Damage Mitigation Measures

Table II shows the percent of people who took specific flood damage mitigation measures at the time of the survey, and if people were flooded in the past, whether this measure was implemented before or after the last flood that she or he experienced. Hurricane Sandy was experienced by almost all (95%) of those who experience flood damage in the past. The mitigation measures are often implemented before the last flood that the respondent experienced. This finding should be interpreted carefully since people can still have implemented these measures in response to a previous flood. Questions with closed answer categories asked whether people implemented the measures listed in Table II. An open‐ended question revealed that these categories of measures capture almost all flood damage reduction measures that our respondents had taken.^13^

**Table II:** Percentage of Respondents Who Implemented a Specific Flood Damage Mitigation Measures and Whether This Measure Was Implemented Before or After the Last Flood that She or He Experienced

	% of Respondents Who Took the Measure	Before (After) Last Experienced Flood
Elevate lowest floor above expected flood level	16%	81% (17%)
Dry flood‐proofing measures		
Water‐proofed walls	31%	66% (34%)
Installed pump or drainage system	46%	85% (15%)
Flood shields or sand bags	32%	79% (21%)
Wet flood‐proofing measures		
Flood‐resistant building materials	33%	59% (41%)
Water‐resistant floor	31%	56% (43%)
Electrical or heating systems above potential flood levels	39%	63% (36%)
Move expensive contents away from flood‐prone parts of the home	49%	57% (42%)

Elevation of homes is implemented by only 16% of the respondents. Even though this measure is very effective in preventing flood damage, it also is one of the most expensive flood risk reduction measures for existing buildings, as indicated in Section 2.2 (see Table I).

Sixty‐nine percent of our total respondents took at least one dry flood‐proofing measure. This is a much higher percentage than those who elevated their houses, which is not surprising since dry flood‐proofing existing buildings is cheaper and more cost effective, as detailed in Table I. Of our total number of respondents, 77% took at least one wet flood‐proofing measure, which is slightly more than for the dry flood‐proofing measures that are also a bit more expensive and less cost effective per home (Table I).

The specific dry flood‐proofing measures, which aim to keep flood water out of a building, that people took are installing water‐proofed walls, a pump or drainage system, and flood shields or sand bags. Water‐proofing walls is the least taken measure and is done by less than one‐third of the respondents. Approximately half of the respondents have installed a pump or drainage system, which is slightly less expensive.

The wet flood‐proofing measures that people took to minimize damage once flood water has entered the building include flood‐resistant building materials, a water‐resistant floor, installing electrical or heating systems above potential flood levels, and moving expensive contents away from flood‐prone parts of the home. Almost half of the respondents moved their contents away from flood‐prone parts of the house, a significantly higher percentage than any of the other measures, presumably because it normally only costs time not money and is usually undertaken once there is an immediate threat of flooding. The other measures require investing in structural adjustments to the building that may not be feasible after a warning and also involve upfront costs. Compared to other measures, a large proportion (43%) of the water‐resistant floors were installed after a flood occurred, which suggests that many people replace floors that were damaged during a flood with water‐resistant floor types, which involve very low additional costs (if any).

### 3.2 Statistical Models of Factors of Influence on Implemented Flood Risk Mitigation Measures

We will now describe the results of the models characterizing the factors that determine whether to elevate one's home or implement dry and wet flood‐proofing measures.

#### 3.2.1 Determining Whether to Elevate One's Home

Table III shows the probit model results for determining whether to elevate one's home. The FEMA flood zone and flood experience variables are insignificant (not supporting H1 and H2). Of the three coping appraisal variables (perceived response efficacy, perceived self‐efficacy, and perceived response costs), the only statistically significant effect is perceived self‐efficacy (partly supporting H3). This implies that homeowners who state they are able to elevate their home are more likely to have done so. Intuitively this makes sense as elevating one's home is a very complicated and time‐intensive mitigation activity to undertake. Expectations of receiving federal disaster relief significantly influence the home elevation decision negatively, thus supporting H8. Note that the magnitude of this effect is small so that a household expecting to receive disaster relief on the order of 50% of experienced flood damage only reduces the likelihood that it will elevate its home by 5%. Whether the home was subject to the NYC building code policy with elevation requirements during construction is positively related to the probability that the home is elevated. Moreover, having a high trust in NYC flood risk management policy in which these building codes form a key element has a positive effect on home elevation (supporting H9). These findings, as well as the insignificance of behavioral characteristics, like risk aversion, discounting, and private values of being well prepared, suggest that home elevation is mainly driven by meeting building code requirements, instead of these behavioral motivations (not supporting H4–H6). The social norm variable is statistically significant with an opposite sign as expected in the full model, but this effect is not robust and disappears when nonsignificant variables are excluded (supporting H7).

**Table III:** Probit Model Results of Home Elevation

	Full Model (1)	Full Model (2)	Significant Only Model
	Marginal Effect	Marginal Effect	Marginal Effect
FEMA 1/100‐year flood zone	0.0215	0.0214	n.a.
Flood experience	0.0220	0.0326	n.a.
High perceived response efficacy	0.0369	0.0402	0.0387
High perceived self‐efficacy	0.2145***	0.2313***	0.2171***
High perceived response costs	−0.01710	−0.0409	−0.0142
Basement	−0.0243	−0.0185	n.a.
Subject to elevation building code	0.0959***	0.1000***	0.1033***
Trust in NYC flood risk management	0.0340	0.0380	0.0597**
Expected federal disaster relief	−0.0008	−0.0008	−0.001**
High discount rate	0.0234	0.0288	n.a.
Private value of preparing for floods	0.0032	n.a.	n.a.
Social norm of preparing for floods	n.a.	−0.0825**	n.a.
Low risk aversion	0.0205	0.0364	n.a.
Age	−0.0022**	−0.0022**	−0.0020**
Female	0.0280	0.0250	n.a.
High education	−0.0343	−0.0440	n.a.
Number of observations	718	695	888
Chi‐square	76.81***	83.41***	92.19***
Pseudo *R* ^2^	0.12	0.13	0.12

n.a. stands for not applicable. ^*^, ^**^, ^***^ indicate significance at the 10%, 5%, and 1% levels, respectively.

Age is the only socioeconomic variable that is statistically significant and it has a negative effect on the decision to elevate one's home. A possible explanation is that older people are less able or willing to elevate their home, for example, because elevated homes are less easily accessible if the front door has to be reached by stairs. Older homeowners do not expect to live in their property for a long period of time and hence cannot justify the investment, and may be less likely to have a mortgage on their home so they are not required to purchase flood insurance even if they reside in the 100‐year floodplain. If they are uninsured, they will not experience the benefit of reduced premiums by elevating their home.

#### 3.2.2 Model Result for Dry Flood‐Proofing

Table IV shows the results of a Poisson model of the number of dry flood‐proofing measures people have taken. The FEMA flood zone variable is positive and significant, which suggests that individuals have taken more dry flood‐proofing measures in the FEMA 1/100 than the low‐risk flood zone (supporting H1). Flood experience is insignificant (not supporting H2). Of the three coping appraisal variables, we find positive significant effects of the perceived response and self‐efficacy variables, but no significant effect for perceived response cost (supporting H3 only for perceived response and self‐efficacy). The coefficient of the high discount rate variable is significant and negative, which implies that individuals with a high discount rate are less likely to invest in dry flood‐proofing measures (supporting H4). This result is expected since these measures can be characterized as an investment with high upfront costs, which pay off in the (perhaps far) future when a flood occurs. Such an investment becomes less attractive when it is evaluated with a higher discount rate. As an illustration, Aerts et al. (2014) show that benefit–cost ratios of dry flood‐proofing a building in the 1/100‐year flood zone with 2 ft above the ground floor level are between 1.04 and 1.21 under a high discount rate of 7%, while these benefit–cost ratios increase to 2.29 up to 2.65 under a lower discount rate of 4%.^14^ A negative effect is found if individuals have a low degree of risk aversion (supporting H5). The effects of the private value and social norms variables, as well as expectations of federal disaster relief, are insignificant (not supporting H6 and H8, supporting H7). Individuals with a basement are more likely to have taken dry flood‐proofing measures, as is expected since these homes are more susceptible to suffering damage in case of flooding than those without a basement and have higher benefits of flood‐proofing. Individuals subject to the elevation requirements in the NYC building codes are less likely to take dry flood‐proofing measures, which suggests these measures are viewed as substitutes for elevation.

**Table IV:** Poisson Model Results of Implemented Dry Flood‐Proofing Measures

	Full Model (1)	Full Model (2)	Significant Only Model
	Coefficient	Coefficient	Coefficient
FEMA 1/100‐year flood zone	0.1453**	0.1487**	0.1182*
Flood experience	0.0864	0.1048	n.a.
High perceived response efficacy	0.1059	0.0750	0.1208*
High perceived self‐efficacy	0.5001***	0.4800***	0.5163***
High perceived response costs	0.1122	0.1273*	0.0840
Basement	0.4041***	0.3933***	0.3972***
Subject to elevation building code	−0.1812**	−0.1471*	−0.158***
Trust in NYC flood risk management	−0.0491	−0.0679	n.a.
Expected federal disaster relief	0.000007	0.0002	n.a.
High discount rate	−0.1358*	−0.1405*	−0.1301**
Private value of preparing for floods	−0.0377	n.a.	n.a.
Social norm of preparing for floods	n.a.	−0.0752	n.a.
Low risk aversion	−0.1790**	−0.1667**	−0.1382**
Age	−0.0027	−0.0026	n.a.
Female	−0.0008	−0.0007	n.a.
High education	−0.0300	−0.0688	n.a.
Number of observations	738	713	833
Log likelihood	−937	−910	−1,049
Chi‐square	114.60***	104.39***	123.96***
Pseudo *R* ^2^	0.06	0.05	0.06

n.a. stands for not applicable. ^*^, ^**^, and ^***^ indicate significance at the 10%, 5%, and 1% levels, respectively.

#### 3.2.3 Model Result for Wet Flood‐Proofing

Table V shows the results of a Poisson model of the number of wet flood‐proofing measures people have taken. Of the FEMA flood zone variables, the 1/100 flood zone is positive significant, which means that homeowners in that zone take more wet flood‐proofing measures than homeowners in the low‐risk flood zone (supporting H1). Similar to the model of dry flood‐proofing, we find positive significant effects of the perceived response and self‐efficacy variables, but no significant effect for perceived response cost (partly supporting H3). The discount rate variable is insignificant (not supporting H4). A negative coefficient is observed for having a low degree of risk aversion (supporting H5). The coefficient of the private value of preparing for floods variable is significant and positive, which implies that individuals with a strong private value of preparing for flooding are more likely to invest in wet flood‐proofing measures (supporting H6). The external social norm and expectations of receiving federal disaster relief variables have insignificant coefficients (supporting H7, but not H8). Individuals with a basement are more likely to have taken wet flood‐proofing measures, as expected. Of the socioeconomic variables, only education level has a positive significant effect on the implementation of wet flood‐proofing measures.

**Table V:** Poisson Model Results of Implemented Wet Flood‐Proofing Measures

	Full Model (1)	Full Model (2)	Significant Only Model
	Coefficient	Coefficient	Coefficient
FEMA 1/100‐year flood zone	0.1402**	0.1209*	0.1269**
Flood experience	0.1161*	0.1125	n.a.
High perceived response efficacy	0.3855***	0.3606***	0.3605***
High perceived self‐efficacy	0.4244***	0.4213***	0.4496***
High perceived response costs	0.0547	0.0599	0.0545
Basement	0.1907***	0.2061***	0.1457***
Subject to elevation building code	0.0521	0.1068	n.a.
Trust in NYC flood risk management	−0.0412	−0.0143	n.a.
Expected federal disaster relief	0.00002	0.0001	n.a.
High discount rate	−0.0738	−0.0686	n.a.
Private value of preparing for floods	0.0982	n.a.	0.1694**
Social norm of preparing for floods	n.a.	0.0442	n.a.
Low risk aversion	−0.1197*	−0.1153*	−0.1244**
Age	−0.0012	−0.0016	n.a.
Female	0.1015	0.0991	n.a.
High education	0.2157***	0.1742**	0.2477***
Number of observations	738	713	864
Log likelihood	−1055	−1021	−1244
Chi‐square	140.37***	127.93***	156.49***
Pseudo *R* ^2^	0.06	0.06	0.06

n.a. stands for not applicable. ^*^, ^**^, and ^***^ indicate significance at the 10%, 5%, and 1% levels, respectively.

## 4 DISCUSSION

### 4.1 Discussion of Main Findings in Relation to Our Hypotheses and Other Studies

Here, we discuss our main findings in relation to the hypotheses that were identified in Section 2.3, of which a summary is given in Table VI.

**Table VI:** Summary of Results of Hypotheses

#	Description	Result
H1	Individuals who live in high FEMA flood risk zones take more flood damage mitigation measures than individuals in low FEMA flood risk zones	Supported for dry and wet flood‐proofing measures
H2	Individuals who experienced a flood in their home have taken more flood damage mitigation measures than individuals without flood experience	Not supported
H3	A high response efficacy, a high self‐efficacy, and low response cost are positively related with the implementation of flood damage mitigation measures	Supported for self‐efficacy for all measures, and response efficacy for dry and wet flood‐proofing measures; not supported for response cost
H4	Individuals with a high discount rate take less flood damage mitigation measures	Supported for dry flood‐proofing measures
H5	Individuals with a low level of risk aversion are less likely to take flood damage mitigation measures	Supported for dry and wet flood‐proofing measures
H6	A strong private value of being well prepared for flooding is positively related with implemented flood damage mitigation measures	Supported for wet flood‐proofing measures
H7	We hypothesize that a strong social norm of being well prepared for flooding is not significantly related with implemented flood damage mitigation measures	Supported
H8	High expectations of receiving federal disaster relief are negatively related with the implementation of flood damage mitigation measures	Supported for home elevation
H9	Those with high trust in NYC flood risk management are more likely to have elevated their home above potential flood water levels	Supported

Our findings confirm H1 that individuals who live in areas with a higher flood risk according to the FEMA flood zone classification take more flood damage mitigation measures than individuals in low‐risk zones. A significant effect of the FEMA flood zone(s) was found in the statistical models for dry and wet flood‐proofing measures, but not for home elevation. It should be noted that home elevation is significantly influenced by whether the home is subject to elevation requirements in the NYC building code when it was built, which only applies to the high‐risk flood zone. Hence, it is not surprising that there is no additional explanatory power of the high‐risk flood zone variable in that model. Our observation that individuals take more flood risk mitigation measures in the high‐risk FEMA flood zone is consistent with other research, which has estimated that such measures are more likely to be cost effective in areas with a high flood probability of at least 1 in 100 (e.g., Aerts et al., 2014; Kreibich, Christenberger, & Schwarze, 2011). Aerts et al. (2014) find positive benefit–cost ratios for elevating homes when they are newly constructed and for dry and wet flood‐proofing buildings in the 1/100‐year flood zone in NYC, but not in the 1/500‐year flood zone where these measures are not cost effective.

Another explanation is that people in the 1/500‐year flood zone may not take flood‐proofing measures because they simplify the risk to the extent they think they do not have a flood problem (Meyer & Kunreuther, 2017). Since people who live in the 1/500‐year flood zone do not face requirements to purchase flood insurance, they may think flood risk is not relevant for them and there is no need to implement risk mitigation measures.

Our findings do not strongly support H2 that individuals with flood experience implement more risk mitigation measures. This finding differs from findings in previous empirical studies that indicate flood experience is an important determinant of the implementation of risk mitigation measures by individuals in samples with a large heterogeneity in flood experiences (e.g., Bubeck, Botzen, Kreibich, & Aerts, 2012b; Osberghaus, 2017; Poussin et al., 2014). This may be due to our special sample where all faced a threat of flooding from Hurricane Sandy and the large majority was flooded during the hurricane, meaning there is little diversity in flood experiences.

H3 stating that a high response efficacy, a high self‐efficacy, and low response cost are positively related with the implementation of flood damage mitigation measures is partly confirmed by our results. A high perceived response efficacy is significantly related with a more frequent implementation of dry and wet flood‐proofing measures, but not with elevation. This means that the perceived effectiveness of dry and wet flood‐proofing plays an important role in the decision to implement these measures, while this is less important for the elevation of homes that appears to be largely driven by building code regulations. A high perceived self‐efficacy significantly influences the implementation of all three categories of measures that we study here, which suggests that the perceived ability to implement flood damage mitigation measures is an important determinant of taking these measures. Perceived response cost, which refers to the dollar costs and time and effort that need to be put in implementing the measures, is not significant in our regression models.

It should be noted that previous studies have measured coping appraisals in different ways, which makes it difficult to compare their results with ours. For instance, Glenk and Fischer (2010) measure coping appraisals as trust in the government, and find that this positively relates with demand of Scottish households for policies to reduce flood risk. This finding about their trust measure is closest to our finding that trust in NYC flood risk management positively relates with the elevation of homes (which supports H9). Grothmann and Reusswig (2006) combine three coping appraisal indicators that are similar to ours in a single index and find it has an important influence on flood preparedness by German households. Our measurement of the three separate coping appraisal variables is similar to the approach taken in Bubeck et al. (2013), who find consistent results to ours: namely, that the implementation of various flood risk mitigation measures by a different sample of German households than Grothmann and Reusswig (2006) is significantly related with perceived self‐efficacy and response efficacy, but not with perceived response cost. Poussin et al. (2014) measured perceived response cost qualitatively using categories of the pure monetary cost of implementing flood risk mitigation measures, and found this is negatively related with the implementation of various flood risk mitigation measures in France. This different finding from ours and that of Bubeck et al. (2013) suggests that the way this variable is measured may influence results, namely, that perceptions of the monetary costs negatively relate with the implementation of flood damage mitigation measures, but the broader measure that also includes perceived time and effort does not. Future research can examine this in more detail.

Overall, it should be noted that behavioral characteristics, like risk aversion, discounting, and private values (H4–H6), are not significantly related to the decision on whether to elevate one's home, which appears to be driven by existing building code regulations, but do influence the unregulated flood‐proofing measures.

H4, that individuals with a high discount rate take less flood risk mitigation measures, is supported for dry, but not for wet, flood‐proofing measures. This difference may be explained by the fact that all our dry flood‐proofing measures entail upfront monetary costs of which the benefits consist of future reduction in damage when a flood occurs, while this is not the case for all wet flood‐proofing measures, which also include moving furniture to higher floors during a threat of flooding. Moving furniture does not entail direct monetary costs since it only takes time, and the reward in terms of lower flood damage is more immediate if it is done when a flood is imminent.

The high upfront costs of dry flood‐proofing measures (Table I) can loom larger for individuals with high discount rates since the future risk reduction benefits are now lower than they would be with lower discount rates. Reynaud et al. (2013) also examine the influence of discounting in a protection motivation theory framework, and find that individual discount rates negatively relate to the number of flood risk mitigation measures that are implemented by farmers in Vietnam.

H5 is supported for dry and wet flood‐proofing measures for which we find a positive relation between the implementation of these measures and the level of risk aversion, i.e., people with a low risk aversion are less likely to invest in these measures. A similar finding was observed by Poussin et al. (2014), who found that more risk‐averse households in France were more likely to implement structural flood risk mitigation measures.

H6, stating that a strong private value of being well prepared for flooding is positively related with implemented flood damage mitigation measures, is confirmed for wet flood‐proofing measures. In this respect, it is important to note that the external social norm variable, which captures the expected approval or disapproval of others for preparing for flooding, is not statistically significant, which supports H7. These findings are consistent with those observed in another context by Viscusi et al. (2011), who showed that private values, but not social norms, influence recycling behavior in the United States. It should be noted that implementing flood risk mitigation measures in one's home is less obvious to others compared to recycling, which makes it more difficult to establish a social norm for these measures.

Expectations of receiving federal disaster relief are significant in the model with only significant variables for the elevation of homes, which supports H8 for that particular measure, but a significant relation was not observed with the implementation of wet or dry flood‐proofing measures. Of these measures, elevation is the most costly (Table I), and the results suggests that individuals are not willing to incur such costs if they expect that the federal government will provide generous compensation for damage in case a future flood occurs. These considerations appear to play a less important role in decisions about the cheaper flood‐proofing measures. Our finding of a negative relation between federal disaster relief and home elevation is consistent with other evidence that shows there is a negative impact of federal disaster relief on flood insurance purchases in the United States (Kousky et al., 2018). Similar negative effects of *ad hoc* government compensation on demand for flood risk mitigation measures and flood insurance have also been observed in studies for other countries, like the Netherlands (Botzen, Aerts, & van den Bergh, 2009; Botzen & van den Bergh, 2012).

### 4.2 Policy Implications

While we have been focused on the likelihood of implementation of flood damage mitigation measures by homeowners, another complementary way for residential property owners to manage flood risk is through the purchase of flood insurance. While the purchase of flood insurance does not eliminate or reduce the probability of a flood event occurrence, or even the actual damage incurred if an event does occur, it transfers the risk of financial loss to a third party, covering the costs of a portion of the incurred damage. But more importantly for our investigation of flood risk mitigation here, insurance can also provide incentives for risk reduction by acting as a price signal of risk and by providing premium reductions to policyholders who protect their property against disaster damage.

In the United States, the majority of residential flood insurance policies are purchased through the National Flood Insurance Program (NFIP) where single‐family residential property owners can purchase up to $250,000 in building coverage as well as an additional $100,000 of contents coverage. The NFIP has developed Flood Insurance Rate Maps (FIRMs) for all NFIP‐participating communities to set risk‐based insurance premiums for each $100 of property (building and content) coverage purchased. These NFIP risk‐based insurance premiums are primarily driven by two key components of the FIRM: the location of the structure in a particular flood hazard area designated on the FIRM, as well as the structure's lowest floor elevation in relation to the FIRM's BFE (NRC, 2015). Consequently, risk‐based premium reductions are primarily driven through these two rating components (e.g., elevate your home above the BFE and the associated insurance premium decreases substantially), and the NFIP does not give premium reductions for other flood risk mitigation measures beyond elevation. But while structural elevation in relation to the BFE therefore can significantly reduce insurance premiums, as well as being recognized as an effective measure for reducing flood risk overall, there are three main issues: (1) elevation of homes is often a least preferred alternative for property owners as was evidenced by our survey data (Table II), while no premium reductions are given to policyholders who take more popular flood‐proofing measures; (2) oftentimes, it is not feasible for property owners to elevate residential structures due to the inherent structural characteristics of the property; and (3) NFIP premiums, and hence also premium reductions for elevation, often do not adequately reflect the risk, as indicated in a recent study of homes in North Carolina (Kunreuther et al., 2018). FEMA is now addressing this problem by updating its maps to more accurately reflect the flood risk that structures face.^15^ Moreover, premiums reflect current risk, meaning that any premium reductions for mitigation do not reflect the benefits of mitigation in a future climate.

From a policy perspective, these aspects have begun to be addressed through the 2014 Homeowner Flood Insurance Affordability Act, whereby alternative mitigation methods to reduce flood risk to residential buildings that cannot be elevated were to be identified, as well as to inform property owners about how these alternative mitigation methods may affect flood insurance premium rates (FEMA P‐1037, 2015). Four wet flood‐proofing techniques were identified—flood openings, elevate building utilities, flood‐proof building utilities, and flood‐damage‐resistant materials—and three dry flood‐proofing techniques—dry flood‐proofing system, floodwall with or without gates, and levee with or without gates (FEMA P‐1037, 2015). While five of these seven techniques are determined to provide moderate or high potential for reducing flood damage (and also likely more cost effective), only flood openings and elevate building utilities provide any potential to lower insurance premiums^16^ (FEMA P‐1037, 2015). Given that we find that individuals who live in areas with a higher flood risk according to the FEMA flood zone classification take more wet and dry flood‐proofing mitigation measures than individuals in low‐risk zones, we would advocate that this uptake could be increased further if insurance premium reductions were made available for these techniques. The NFIP could monitor whether these structural adjustments are in place, and hence determine whether the policyholder is eligible for a premium discount, but it should be noted that this may be less feasible with other risk mitigation measures, like moving contents to higher floors. The premium reductions can be combined with making low‐interest mitigation loans available to individuals in flood‐prone areas, which spread mitigation costs over time, and that way overcome individual myopia and high time discounting (Meyer & Kunreuther, 2017).

Our results also suggest that high response efficacy and high self‐efficacy beliefs by property owners are positively related with the implementation of wet and dry flood‐proofing mitigation measures. Therefore, an alternative to providing insurance premium reductions to increase uptake of these risk reducing measures would be to tailor the information concerning these mitigation techniques. That is, an information campaign aimed at increasing the uptake of wet and dry flood‐proofing measures could focus on the effectiveness of these measures as well as how they can be (easily) implemented, as opposed to only the threat appraisal components of the flood risk. Moreover, risk awareness of individuals in flood‐prone areas may be improved if flood risks are not communicated to them in annual flood probabilities, as FEMA currently does, but if instead flood probabilities are communicated over a longer time, such as 30 years (Kunreuther, 2018).

However, with regard to elevation, we do find evidence that high expectations of receiving federal disaster relief are negatively related with its implementation. In fact, 32% of our respondents expect to receive federal disaster relief if their home were flooded in the future, and these individuals believe they will receive significant compensation (51% on average) to cover their flood damage. Unfortunately for property owners, research (Kousky et al., 2018) has shown that average individual assistance grants are actually quite small—approximately $3,000—not nearly enough to cover the significant costs of flooding. Providing more detailed information concerning the typical disaster assistance provided to property owners as opposed to the larger values given to communities could help to assuage this affect.

## 5 CONCLUSION

Improving our understanding of why some people in flood‐prone areas prepare well for flooding and implement flood damage mitigation measures, while many others are unprepared, is important for the design of policies to help people make better flood preparedness decisions. This study offers insights into the implementation of flood damage mitigation measures by homeowners who live in flood‐prone areas in NYC. We make use of survey data about implemented flood damage mitigation measures by more than 1,000 homeowners and factors of influence on taking these measures. Our theoretical basis for explaining flood preparedness decisions is protection motivation theory, which explains individual decisions about preparing for risk on the basis of threat appraisal and coping appraisals. We extend this framework using other variables that can have an important influence on individual decision making under risk, such as risk attitudes, time preferences, social norms, trust, building code regulations, and expectations of receiving federal disaster assistance.

Our findings in relation to our main hypotheses can be summarized as follows. Individuals who live in high flood risk zones take more flood‐proofing measures in their home than individuals in low‐risk zones, which suggests these former people have a higher threat appraisal. With regard to the coping appraisal variables, we find that a high response efficacy and a high self‐efficacy play an important role in taking flood damage mitigation measures, while perceived response cost is not significantly related to the implementation of these measures. In addition, a variety of behavioral characteristics influence individual decisions to flood‐proof their homes, such as risk attitudes, time preferences, and private values of being well prepared for flooding. These behavioral variables are not significantly related with whether individuals elevate one's home, which is mainly influenced by building code regulations as well as trust in flood risk management policies that advocate elevation. Moreover, decisions to elevate are negatively related with expectations of receiving federal disaster relief.

We discuss a variety of policy recommendations to improve individual flood preparedness decisions and increase the uptake of cost‐effective flood damage mitigation measures. Improved incentives for investing in risk mitigation can be given through flood insurance by charging premiums that better reflect risk, and by offering risk mitigation loans to spread the investment costs over time. Premium reductions should be given to policyholders who mitigate risk that reflect the risk reduction, which can be achieved from elevation as well as other flood‐proofing measures. Communication campaigns could focus on the effectiveness of flood damage mitigation measures as well as how they can be (easily) implemented. Risk awareness can be improved by communicating about flood risk over a longer time horizon, such as 30 years, instead of the common practice to inform individuals about annual flood probabilities.

This study is a step forward in improving our understanding of individual flood preparedness decisions by identifying a range of behavioral motivations for taking flood damage mitigation measures. Our findings are derived from a particular sample of homeowners in flood‐prone areas in NYC, so it will be useful to examine whether similar behavior is observed in other flood‐prone areas in the United States that have not experienced a severe disaster such as Hurricane Sandy. Moreover, future research is needed to test the effectiveness of our recommendations to increase investments in cost‐effective flood risk mitigation measures, for example, using economic experiments. In particular, a combination of improved communication about risk and mitigation measures with economic incentives to address behavioral biases seems to be the most promising avenue for future research.

## Supporting information


**Table AI**. Description and Coding of the Dependent and Explanatory Variables Used in the Statistical Models

## Footnotes

1
https://www.munichre.com/topics-online/en/2017/12/hurricane-harvey

2 The location of the non‐NYC residents who participated in the pretest was not recorded. The purpose of pretesting the survey with these respondents was for the interviewers to practice the questionnaire and check whether questions were clear to respondents. The pretest data were not used in the final analysis.

3 Note that these detailed cost–benefit analyses results are reported in the supplementary material by Aerts et al. (2014) and not in the text of their main paper.

4 This ambiguity arises because while high flood risk perceptions may be a reason for taking flood‐proofing measures, these risk perceptions may decline again after people have taken measures to reduce their risk. Cross‐section survey data of implemented flood‐proofing measures only observe risk perceptions after the protective measures have been adopted so that the role of risk perceptions in the decision to take these measures cannot be identified. We overcome this identification problem by including an indicator of objective flood risk, namely, the FEMA flood zone, which may relate to threat appraisal if people in a higher‐risk flood zone perceive they are at a high risk of flooding. Although we cannot rule out that such a problem with identifying causality also occurs with the coping appraisal variables, we expect that the change in risk perception following the implementation of risk reduction measures is stronger than a possible change in coping appraisals.

5 The U.S. Federal Emergency Management Agency (FEMA) is charged with mapping flood risk in hazard‐prone areas and releasing this information to the public. Different FEMA flood zones correspond to different levels of risk. Usually, high‐risk areas are defined as those where there is a higher than 1% chance of being flooding in any given year.

6 For instance, this proxy does not capture the behavior of individuals with a high threat appraisal who may have migrated out of the flood‐prone area. In other words, those with low threat appraisal are the ones who remain in the flood zone. It also does not identify people who may have a low threat appraisal because they expect the government to provide protection to reduce damage in well‐specified flood plains. However, we expect these effects to be minor since previous research has shown there is mainly inward migration in the NYC flood zones because these areas are perceived to be attractive places in which to live (Aerts & Botzen, 2012). Moreover, the government has not undertaken substantial investments in flood prevention in 1/100‐year flood zones because otherwise these areas would not have been classified by FEMA as being a 1/100‐year zone (protected areas are mapped to be out of these high‐risk flood zones).

7 Seventy‐four percent of our respondents were flooded in the past.

8 We replaced the flood experience variable with two variables: the amount of damage suffered during the last experienced flood and estimated inundation level at the respondent's census block during an extreme flood event like Sandy. We obtained this latter variable from the flood risk analysis done for NYC by Aerts, Lin, Botzen, Emanuel, and de Moel (2013b). Both variables turned out to be insignificant, and their inclusion did not affect the main relationships of interest (detailed results not shown here).

9 These variables are included separately since model fit is better that way than including them as interactions or a combined variable of high coping appraisals.

10 This variable serves as a proxy for whether individual homes were subject to the elevation requirements in the NYC building code when they were constructed, but we cannot determine whether individuals elevated their home voluntarily or mandatorily. Homes are required to be elevated when a building is renovated or repaired for a cost of 50% or more of the value of the home but we expect this to occur rarely. These regulations are often avoided by undertaking several small cost renovations over time, so that each renovation costs less than 50% of the building value (Aerts & Botzen, 2011).

11 An exception to this step‐wise exclusion is made for the three coping appraisal variables *Ci* (explained in Section 2.3) that were always kept in the model estimations because, according to protection motivation theory, it is this combined group of variables that significantly influences protective behavior.

12 “Don't know” and “not sure” responses to questions are coded as missing observations for the dependent and explanatory variables in our analysis.

13 An open‐ended question asked whether the respondent took any other measures to reduce flood risk. Most of the answers to these questions repeat the measures that people already indicated to have implemented that were listed in the close‐ended question in Table II. But a few answered with new flood risk reduction measures; six people indicated to have built a flood wall around their house. This very small number of other flood risk reduction measures mentioned by people suggests that the measures listed in Table II cover the large majority of measures that people took.

14 These are the results from Aerts et al. (2014) for a middle climate change scenario and most likely risk estimates.

15
http://www.rms.com/blog/2018/06/27/a-turning-tide/

https://www.casact.org/community/affiliates/cagny/0518/Hayes.pdf

16 Flood openings is not a wet flood‐proofing measure we ask survey respondents about, although elevate utilities is.
