# Supplementary material for: Adoption of Individual Flood Damage Mitigation Measures in New York City: An Extension of Protection Motivation Theory
Source: Risk Anal. 2019 Apr 25;39(10):2143–59. doi: 10.1111/risa.13318 (PMC6850606; doi:10.1111/risa.13318)
Supplement: Supplementary file 1 — Table AI. Description and Coding of the Dependent and Explanatory Variables Used in the Statistical Models [file RISA-39-2143-s001.docx]

**ONLINE APPENDIX A. CODING OF VARIABLES**

*Table AI. Description and coding of the dependent and explanatory variables used in the statistical models*

| Dependent variables : |  |
| --- | --- |
| Elevation | Binary variable, 1=respondent has elevated her/his home above the expected water level of a flood, 0=otherwise |
| Dry flood-proofing measures | Count variable of the number of dry flood-proofing measures a respondent has implemented |
| Wet flood-proofing measures | Count variable of the number of wet flood-proofing measures a respondent has implemented |
| Explanatory variables |  |
| FEMA 1/100 year flood zone | 1= respondents lives in the FEMA 1/100 year flood zone, 0=otherwise |
| Flood experience | 1=respondent has been flooded in the past, 0=otherwise |
| High perceived response-efficacy | 1=respondent answers very effective or effective to a question that asks how effective she/he thinks the action (either elevation, dry or wet flood-proofing) is in protection their home and its contents against flooding, 0=somewhat effective or not at all effective |
| High perceived self-efficacy | 1=respondent answers definitely are able or possibly able to a question that asks to what extent are you or a member of your household able, to actually carry out these measures (either elevation, dry or wet flood-proofing), 0=possibly not able, or definitely not able |
| High perceived response costs | 1=respondent answers very high or high to a question that asks how much the total costs (including dollar costs and time and effort) would be to take these measures (either elevation, dry or wet flood-proofing)?, 0=somewhat high or not high at all |
| Basement | 1=respondent has a basement, 0=otherwise |
| Subject to elevation building code | 1=home of respondent is built after 1986 and hence subject to the NYC elevation building code regulation, 0=otherwise |
| Trust in NYC flood risk management | 1=trust government’s flood risk management somewhat or completely, 0=does not trust them very much or at all |
| Expected federal disaster relief | Percentage of damage a respondent expected to be compensated by the federal government in case a flood occurs |
| High discount rate | 1= respondent answered a score 1-5 on the scale of the question “When it comes to financial decisions, how would you assess your willingness to give up something today in order to benefit from that in the future?” 1= completely unwilling to give up something today and 10= very willing to give up something today, 0=otherwise |
| Private value of preparing for floods | 1= respondent agrees or strongly agrees with the statement “I would be upset if I noticed that someone who got flooded was insufficiently prepared for flooding and needed to request federal compensation for flood damage he suffered”, 0=respondent disagrees, strongly disagrees or neither agrees nor disagrees |
| Social norm of preparing for floods | 1= respondent agrees or strongly agrees with the statement “Other people would be upset if they noticed that someone who got flooded was insufficiently prepared for flooding and needed to request federal compensation for flood damage he suffered”, 0=respondent disagrees, strongly disagrees or neither agrees nor disagrees |
| Low risk aversion | 1= respondent answered a score 1-6 on the scale of the question “Using a 10-point scale where 1 means you are not willing to take any risks and 10 means you are very willing to take risks, what number reflects how much risk you are willing to take?”, 0=otherwise |
| Age | Age of the respondent in years |
| Female | 1=respondent is female, 0=respondent is male |
| High education | 1=highest education level is at least college, 0=otherwise |
